# Narratives before numbers: Reimagining conversations about race, ethnicity, and ancestry information in genetic counseling practice

**DOI:** 10.1002/jgc4.70124

**Published:** 2025-11-02

**Authors:** Emily Peugh, Sara Chandros Hull, Leila Jamal

**Affiliations:** ^1^ Clinical Center, Department of Bioethics National Institutes of Health NIH Bethesda Maryland USA; ^2^ Bioethics Core National Human Genome Research Institute, NIH Bethesda Maryland USA; ^3^ Genetics Branch, Center for Cancer Research National Cancer Institute, NIH Bethesda Maryland USA

**Keywords:** bioethics, genetic counseling, narrative medicine, patient‐centered care, practice guidelines, race, ethnicity, and ancestry (REA)

## Abstract

As genomic testing becomes more common, it is essential to re‐examine practical and ethical arguments for and against eliciting race, ethnicity, and ancestry (REA) information from patients as a default practice in genetic counseling. In this article, we evaluate current and historical reasons for using REA information in clinical genetics encounters. We argue that in many, if not most, cases, the value of this practice for patient risk assessment and the establishment of test eligibility is questionable. This does not mean we do not see any value in the practice of discussing REA at all. Rather, we propose that discussions about REA should be patient‐led and relevant to discussions about their experiences, values, and goals. To facilitate this change, we offer some practical reasons for limiting the default practice of collecting REA information in genetic counseling. Additionally, we evaluate the ethical acceptability of this practice, anchoring our ethical analysis in three considerations: (1) the impact of the use of REA information in determining eligibility for genetic testing and assessing genetic risk; (2) the influence of inequitable genomic database representation; and (3) the effect of discussing REA on the therapeutic relationship. Our analysis of these considerations leads us to argue for a patient‐centered, narrative framework that treats the collection of REA information as a way of encouraging patients to articulate the relevance of these identities to their counseling goals, if and in which context they choose.

## 1 INTRODUCTION

The collection and use of information regarding race, ethnicity, and ancestry (REA) in genetic counseling practice has sparked debates about its ethical appropriateness and impact (Cerdeña et al., 2022). Traditionally, genetic counselors have asked patients for information about their REA as part of routine family history discussions. Very few studies have examined how consistently genetic counselors obtain or use this information (Hubbel et al., 2023). Professionals rely on imprecise, one‐size‐fits‐all guidance about how to discuss REA information, how to incorporate responses into decision‐making, and what sociodemographic labels (if any) to use (Hubbel et al., 2023).

In 2023, the National Academies of Sciences, Engineering, and Medicine (NASEM) released a report titled “Using Population Descriptors in Genetics and Genomics Research” (National Academies of Sciences, Engineering, and Medicine, 2023). This report reviewed current methods and examined the advantages, drawbacks, and complexities of using population descriptors linked to concepts of ancestry in human genetics and genomics research. In response to this report, several leading scientific, professional organizations, and journals in human genetics and medicine issued more specific guidelines detailing the proper uses of traditional population descriptors and offering considerations for interpreting genetic ancestry from existing datasets (Mauro et al., 2022). Collectively, these initiatives aim to reduce misconceptions about the connection between genetic ancestry and categories of race and ethnicity reported by patients or clinicians. However, the focus of these efforts has been on genomic *research*, and the appropriateness of these recommendations for clinical practice is not clear.

It is essential to re‐examine arguments for and against the practical value and ethical permissibility of routinely eliciting REA information from patients. We evaluate current and historical reasons for using REA information in clinical genetics encounters, arguing that, in many cases, the practice is of questionable value in the context of modern clinical genetics. Additionally, we examine the *ethical* acceptability of this practice according to three considerations: (1) the impact of the use of REA information in determining eligibility for genetic testing and assessing genetic risk; (2) the influence of inequitable genomic database representation; and (3) the effect of discussing REA on the therapeutic relationship. Drawing on this analysis, we advocate for the practice of narrative medicine to be applied to the field of genetic counseling, reframing the collection of REA information as an invitation for patients to determine whether these identities align with their clinical objectives.

## 2 DEFINITIONS

Sociodemographic data, such as REA are commonly collected as part of clinical encounters (Hubbel et al., 2023). Each of these descriptors has a distinct definition and sociocultural meaning, making the precise and constructive use of descriptors a critical component of inclusive genetic health care (Cho et al., 2022). “Race” refers to a socially constructed group identity that is defined according to phenotypic characteristics such as skin color. The concept of race, which arose as a consequence of racism, is often associated with social status and either self‐identified or assigned class (Jones, 2000). Racial categories have been upheld and institutionalized to reinforce social hierarchies, justify differential treatment, and uphold systems of power and oppression. “Ethnicity” refers to a social group defined by a spectrum of shared behaviors, experiences, and attributes, including language, culture, ancestry, and geographical location (American Anthropological Association, 2016). These two characteristics are typically either self‐identified by patients or ascribed by clinicians in test requisition forms and medical records. When REA information is collected via intake forms, the descriptors provided are often confined to a default list of discrete categories that are not standardized across clinical contexts, grouping patients into reductive categories that do not capture their diverse identities (Popejoy et al., 2020). Where free response options for reporting ancestry exist, there is no evidence or guidance about how to incorporate these into risk assessment or clinical laboratory analyses (Wagner et al., 2023).

Often conflated with race and ethnicity, “ancestry” typically groups people by geographic origins (Mathieson & Scally, 2020). Ancestry information is routinely collected by genetic counselors to help assess the frequency of certain genetic variants within specific populations. There are limited standardized criteria for discussing and utilizing ancestry information in genetic counseling practice, which is complicated by growing population interactions, blurred genetic boundaries, and inadequate options for categorizing individuals (Popejoy et al., 2020; Redman et al., 2024). A recent survey revealed that 96.5% of genetic counselors collect patient ancestry information, 53.2% of which obtain this information only by asking the patient directly and 32.4% obtain ancestry by asking the patient and through intake forms (Hubbel et al., 2023).

In practice, most patients do not have a complete understanding of their ancestral origins (Mersha & Abebe, 2015). As a result of these limitations, integrating ancestral information specifically, and REA information more broadly, into clinical assessments has the potential to both obscure the identities of those from underrepresented ancestral groups and reinforce reductive, socially defined race and ethnicity categories, inadvertently contributing to the re‐inscription of racial biases in medicine and providing a false sense of “precision” in precision health.

## 3 ORIGINS AND PURPOSES OF COLLECTING REA INFORMATION IN GENETIC COUNSELING

The practice of eliciting REA information in genetic counseling began as a way of approximating a patient's genetic origins. Broadly, the purpose of doing this was (and remains) threefold: (1) To estimate the probability that an individual is a carrier for a condition despite a negative genetic test result (i.e., estimating residual risk); (2) To identify individuals whose prior probability of testing positive for a condition is high enough to justify insurance coverage for a test (i.e., establishing test eligibility); and (3) To inform a clinician's test selection by making sure that the variants tested for include those that occur more commonly in an individual's reported ancestral group (i.e., informing test selection) (Hatchell et al., 2025).

### 3.1 Estimating residual risk

Residual risks (RRs) are estimates of the likelihood that an individual carries a pathogenic variant in an autosomal recessive gene after receiving a negative genetic test result for pathogenic variants in that gene. RRs are typically calculated by subtracting the frequency of carriers that can be detected by a test from the estimated frequency of carriers in a given population, which is calculated using the Hardy–Weinberg Law (Hatchell et al., 2025). Limitations of RR estimates include the fact that the incidence of disorders is either inaccurate or entirely unknown for many populations, and that few populations conform to the assumptions of Hardy–Weinberg equilibrium, such as random mating and no inward or outward migration (Nussbaum et al., 2021; Overall & Nichols, 2001). The practice of estimating relative risks (RRs) based on self‐reported or clinician‐reported REA stems from an era when testing was limited to a small number of common variants in a gene at a time.

### 3.2 Establishing test eligibility

Because of economies of scale made possible by next‐generation sequencing (NGS), the cost of clinical genetic testing has decreased since it was first introduced (Pruneri et al., 2021). Nonetheless, many genetic tests remain prohibitively expensive for individual patients without robust insurance coverage. Public and private insurers also have incentives to minimize their coverage costs associated with genetic testing, limiting coverage to tests with a high likelihood of impacting clinical management and outcomes. In addition, clinicians must weigh the potential benefits of a test against its potential harms. For these reasons, REA information is used to evaluate the probability that a patient carries a pathogenic variant in a specific gene or group of genes. Theoretically, the goal of doing this is to improve the yield of testing by prioritizing access for individuals who come from ancestral groups known to have relatively high incidences of certain genetic conditions. The use of REA information to establish test eligibility, and by extension eligibility for insurance coverage, is codified in many practice guidelines including the NCCN guidelines for Hereditary Breast, Ovarian, Pancreatic, and Prostate Cancer (version 2.2025), which specifies that individuals with personal and/or family histories of cancer and Ashkenazi Jewish ancestry (or other ancestries known to be associated with founder variants in *BRCA1* and/or *BRCA2*) should be offered genetic testing.

### 3.3 Informing test selection

REA information can influence a provider's selection of a genetic test by guiding decisions about the type of test, the conditions to test for, and the specific genetic variants to include. These factors are used as proxies for population‐level differences in genetic risk and variant prevalence that are deemed relevant to individual patients based on their self‐reported REA identities. The motivating concern behind this practice is to make sure that targeted panel testing is optimized to detect ancestry‐specific variants believed to be relevant to a patient and/or to alert the clinical laboratory that it should prioritize the sequencing coverage of certain variants.

Specific examples of each of these uses are provided in Table 1. There are additional clinical laboratory uses of REA information not mentioned here that are summarized and critiqued elsewhere (Hatchell et al., 2025). There are additional reasons to discuss REA information in clinical genetics encounters that do not relate to risk assessment, establishing test eligibility or informing test selection. For example, such discussions can deepen counselor's understanding of a patient's background and values or provide space for patients to articulate core narratives related to their identities. Note that in this section of the article, we primarily critique the medical uses of this information.

**TABLE 1:** Examples uses of ancestry information in genetic counseling.

Use case	Rationale	Example conditions and populations	Best practices for inclusive care
Refining reproductive risk estimates	Screen for autosomal recessive conditions that cluster in specific ancestral groups due to bottleneck effects or heterozygote advantage	Tay‐Sachs disease in individuals of Ashkenazi Jewish/French‐Canadian descent, sickle cell disease and other hemoglobinopathies in individuals of African descent, and cystic fibrosis in individuals of European descent (Committee on Genetics, 2017)	Utilize pan‐ethnic expanded carrier screening, as recommended by ACMG and ACOG, rather than testing for common variants solely based on ancestry
Developing a differential diagnosis	Estimate the prior probability that a patient will test positive for a genetic condition when conditions that cluster in specific ancestral groups are suspected	Spinocerebellar ataxia type 3 (SCA3) which arises more commonly in individuals of Portuguese or Azorean descent (Sequeiros et al., 2010)	Refer to ancestry as one of many diagnostic tools while prioritizing clinical presentations
Selecting an appropriate genetic test panel for a patient	Ensure the variants tested for include those which are specific to the ancestral population the patient is from	G115E variant in *HOXB13*, which is prevalent in individuals of West African and Martinican descent (Darst et al., 2022)	Avoid limiting panels to test for common variants based on REA information alone, and instead use comprehensive sequence‐based testing for full coverage as is indicated by current ACMG and NSGC guidelines
Identifying patients that may benefit from genetic testing	Prioritize patients who receive genetic testing services in scenarios where it is not desirable or possible to test every patient	NCCN guidelines state individuals of Ashkenazi Jewish descent with personal or family histories of cancer should be offered cancer susceptibility genetic testing (NCCN Guidelines v1.2026)	Adhere to guidelines and research that advocate for universal testing for hereditary cancers, rather than ancestry‐based eligibility criteria (Esplin et al., 2022)

The effectiveness of using REA information as a proxy for population‐level differences in genetic risk and variant prevalence is limited in all three of these cases by large gaps in what is known about disease incidence and genetic variation in most non‐European ancestral groups and the inconsistency and imprecision of patient‐ or clinician‐reported REA information. These limitations are compounded by the fact that most known populations do not conform to the assumptions of Hardy–Weinberg equilibrium. These shortcomings have become even more apparent as more accurate SNP‐based ancestry testing has become more commonplace (Shraga et al., 2017), and as NGS testing has become the norm. Whole gene sequencing makes it possible to identify most (if not all) variants in a gene or group of genes. From a practical standpoint, it is unclear whether the routine practice of collecting REA information remains justifiable, especially given evidence that clinical laboratories can infer ancestry information from genetic data itself and that these inferences correlate poorly with self‐ and clinician‐reported REA data (Gouveia et al., 2025; Kaseniit et al., 2020). Furthermore, population trends in favor of more mixed ancestries also call this routine practice into question (Lowe et al., 2024).

## 4 ETHICAL CONSIDERATIONS

The practical limitations described above are not the only relevant considerations for evaluating the use of REA data in genetic counseling. Ethical analysis is an additional tool for evaluating the benefits and disadvantages of this practice. Decisions about whether and how to engage in ancestry‐centered conversations with patients are tethered to core principles of biomedical ethics, which are summarized in Table 2. In the following section, we use these principles to analyze ethical concerns raised by using REA information in genetic counseling.

**TABLE 2:** Ethical considerations and principles relevant to uses of REA information in genetic counseling.

	Description	Related ethical principle(s)	Application of principles	Potential benefits and risks
Consideration 1	Impact of the use of REA information in determining eligibility for genetic testing and assessing genetic risk, especially for patients who do not know their precise ancestry	Justice; beneficence/nonmaleficence; veracity (telling the truth)	Genetic counselors ought to act in ways that promote their patient's best interests, ensuring equitable access to testing approaches (beneficence); avoiding harm by excluding patients that have minimal insight into their ancestral heritage (nonmaleficence); and employing fair practices that do not disproportionately affect certain populations (justice). Further, genetic counselors should clearly communicate the limitations and biases of testing criteria (veracity)	Benefits: Supports risk stratification if ancestry‐based data are accurate and equitable Risks: May overlook patients with incomplete or imprecise ancestry knowledge
Consideration 2	Influence of genomic database representation on interpretation of REA information	Justice	To avoid exacerbating underlying knowledge deficits in the clinical encounter, genetic counselors should inform their patients that the genetic makeup of some groups is poorly understood, especially if the patient comes from a group known to be underrepresented in genomics research (e.g., Indigenous communities)	Benefits: May be useful in patients with granular knowledge of their ancestry Risks: May exacerbate existing health disparities resulting from lacking reference data from underrepresented genomes
Consideration 3	Effect of discussing REA and identity on the therapeutic relationship, particularly with patients from underrepresented groups	Beneficence/nonmaleficence; relational ethics/ethics of care	During clinical interactions, genetic counselors should aim to create a trusting relationship with their patients by making certain that inquiring about a patient's REA enhances their well‐being (beneficence); does not jeopardize the therapeutic relationship (nonmaleficence); and is not perceived as culturally insensitive or invasive, thus threatening the relational obligations central to the field of genetic counseling (relational ethics/ethics of care)	Benefits: May foster deeper understanding of patient background, values, and needs: Strengthens therapeutic relationship Risks: Risk of cultural insensitivity, patient discomfort, or misinterpretation if navigated poorly and without trust

### 4.1 Potential impact on those who do not know their precise ancestry

Using ancestry information to establish genetic testing eligibility exacerbates inequitable access to health information, especially for patients who do not know their precise ancestry. Current practice guidelines that use ancestry information to establish RR and testing eligibility are more likely to benefit patients who have the most accurate REA information about themselves and whose ancestral DNA has been well‐studied and cataloged in clinical databases (Shraga et al., 2017). Consequently, patients with less prior awareness of or access to this information will be at a relative disadvantage when asked by a genetic counselor about their ancestry, and there will be variation in the accuracy of the information obtained and reported on a test requisition form. Familiarity with and disclosure of family medical history varies between ethnic groups, as do health communication styles and comfort levels when interacting with healthcare professionals (Hull & Natarajan, 2022; Matalon et al., 2023). Longstanding cultural, historical, and structural barriers to care influence these variations, providing reason to support the collection of REA information as a tool to understand how patients navigate the healthcare system and how access to genetic counseling services might be shaped by larger social circumstances. However, when collected to determine testing eligibility rather than to decrease healthcare inequalities, REA information risks being used to reinforce inequalities instead. Individuals who may lack access to personal ancestry information due to external forces, such as descendants of enslaved people, descendants of communities that faced genocide, those conceived via donor gametes, adoptees, and those whose conception involved displacement or violence, are most affected by these disadvantages. Most populations have at least some degree of interaction with one another, calling into question the accuracy and precision of all self‐reported ancestry information, even among patients who are confident they know where their ancestors came from (Kaseniit et al., 2020). The reliance upon self‐reported ancestry to guide clinical decisions creates barriers to equitable access to the information revealed by genetic testing, thus introducing a risk of overlooking potential carriers of pathogenic variants in genes that profoundly affect healthcare management and outcomes.

### 4.2 Influence of inequitable genomic database representation

The uses of REA information in genetic counseling practice described above unfairly advantage those whose ancestral groups are better understood and represented in genomic databases and genetics research. A majority of genetic studies do not reflect the diversity of the world's populations, favoring European populations and restricting the applicability of genetic risk assessments (Sirugo et al., 2019). Limited representation in genomic databases can lead to imprecise assessments of genetic risk, undermining the very goals of precision healthcare. For instance, if a patient of American Indian descent self‐reports that they are White, the genetic counselor may test them as such, producing “normal” results that appear comprehensive but overlook risks tied to the unrecognized ancestry. This case of poor clinical translation and test optimization is further complicated by the gross underrepresentation of Indigenous communities in genomic research (Claw et al., 2018). The continued use of REA as a basis for determining testing eligibility, coverage, and RR can cause the inequities in genomic research to translate to results in the clinic. While genetic testing that can detect most if not all variants (whether known, unknown, common, or rare) in multiple genes at once is now routine, knowledge about genetic variants remains unevenly distributed, with much of the existing research predominantly focused on populations of European descent (Landry et al., 2018). This imbalance challenges the application of REA‐based clinical practices to individuals with underrepresented or unknown ancestral origins. Reliance upon restrictive and inconsistent ancestry categories also fails to capture the complexity of genetic variations, leading to potential misinterpretations and incomplete assessments of genetic risks.

### 4.3 Effects of eliciting REA information on the therapeutic relationship

Little attention has been paid to the question of whether and why eliciting REA information could be alienating to patients from underrepresented groups. The therapeutic relationship requires genetic counselors to listen closely and nonjudgmentally to their patients and to practice sensitivity in conveying technical information and test results (Biesecker, 2019). If REA information is not elicited in a purposeful and patient‐centered manner, the therapeutic relationship may be damaged. Some patients, especially those from minoritized populations, experience hesitancy and discomfort when healthcare providers ask for REA information, especially when the rationale for asking is not articulated clearly (Kiran et al., 2019). Discussing REA information in a clinical environment can be complicated due to its sensitive nature as well as the lack of thorough education on how to manage these conversations.

Racial discordance between genetic counselors and their patients is likely to heighten this sensitivity, given that 87% of respondents from the National Society of Genetic Counselors (NSGC) 2024 Professional Status Survey self‐identify as White (National Society of Genetic Counselors, 2024). There is evidence that patient–physician dyads that are ethnicity‐concordant are more likely to result in highly rated patient experiences (Takeshita et al., 2020) and that patients from minority groups are generally uncomfortable being asked about their race or ethnicity in healthcare settings unless they understand why (Baker et al., 2007). Therapeutic relationships between genetic counselors and their patients are built on trust, support, and effective communication that together create an open environment for patients to express their questions, concerns, and fears. Centering ancestry in conversations about genetic testing when there are no clear clinical indications can harm the relationship between patient and provider.

## 5 LESSONS FROM NARRATIVE MEDICINE

The ethical analysis in the previous section raises questions about whether, when, why, and how genetic counselors should be eliciting REA information for clinical purposes. In considering this analysis, it is crucial to acknowledge that there are valuable nonclinical reasons for genetic counselors to discuss REA with their patients. Narrative medicine can offer a transformative approach to genetic counseling that supports patients in expressing their multifaceted identities. As an evolving approach to patient‐centered care, narrative medicine was developed to strengthen the relational components of healthcare to generate both clinically and personally meaningful patient outcomes (Kalitzkus & Matthiessen, 2009). When the narrative medicine framework is applied to clinical interactions, healthcare providers use the key components of attention, representation, and affiliation in order to become more familiarized with their patients, find deeper meaning in what is being communicated, and advocate for the patient's best interest (Charon, 2006). Not only can narrative expression be central to a patient's experience with illness, but the process can also inform how they articulate the relationship between their race, ancestry, identity, and self‐perception. Clinicians in all settings must engage in narrative interactions with their patients to establish meaningful relationships and empower autonomous decision‐making.

The model of narrative medicine originates from the ideas of Michael White and David Epston, who developed the approach in the context of family therapy and social work (White & Epston, 1990). Today, narrative medicine draws influences from anthropology, feminism, philosophy, psychology, community work and activism, and justice frameworks (Freedman & Combs, 1996; Madigan, 2019; Russell & Carey, 2004; White, 1997). Narrative practice is a collaborative approach that acknowledges people's lives as composed of multiple, richly detailed stories. By eliciting these stories, genetic counselors can help individuals create new meanings around their experiences with a genetic condition. The goal of this practice is to emphasize that individuals are experts in their own lives, possessing a wealth of skills, experiences, hopes, and values (White, 1997; White & Epston, 1990) that can support their adaptation to living with a genetic condition or its risks. Instead of intervening upon people's experiences or decisions, narrative practice focuses on providing opportunities for individuals to explore, or “externalize” multiple co‐existing perspectives on an issue. This process can uncover a patient's preferences, abilities, and knowledge, creating an opportunity for them to reconnect with overlooked personal or family stories that are not often embraced in traditional clinical settings. These rediscovered narratives empower patients to view themselves in a new light, enabling them to draw on their own strengths to address whatever challenges they face.

For example, migration and differences in medical documentation practices might cause a patient of Trinidadian heritage seeking risk assessment for hereditary cancer to be uncertain how to respond when asked about their family's origins, particularly when their REA information is specifically elicited, given that Trinidadian and other Caribbean populations exhibit high levels of ancestral variation with contributions from West African, European, South Asian, and Indigenous American ancestral components. Furthermore, the region's complex demographic history is shaped by historical trends such as transatlantic slavery, European colonization, and South Asian indentured labor migration. In such a situation, a narrative medicine framework can be used to encourage the patient to share their broader understanding of their family's origins in their own words. Irrespective of the “accuracy” of this narrative, a genetic counselor can glean valuable understanding from it, including a better understanding of the patient's relationships with their relatives and ancestors, feelings about their country's history of colonialism, slavery, and migration, and aspects of their identity which they feel are valuable and important today. This trusting dialogue respects the patient's cultural identity, perspective, and experiences while assisting the genetic counselor in contextualizing possible genetic risk factors.

Despite the theoretical appeal of narrative medicine, few studies have evaluated the use of narrative techniques in genetic counseling. A limited body of research suggests that integrating narrative medicine into genetic counseling can improve patient satisfaction and counselor‐patient relationships by aligning technical information with the patients' lived experiences (Dane et al., 2024). Key outcomes of using narrative approaches in genetic counseling have included improving insight, facilitating support, enhancing communication, and promoting optimism and coping (MacLeod et al., 2018; Mendes et al., 2010, 2013; Peters et al., 2004, 2006, 2012; Spiers et al., 2020; Stopford et al., 2019). Allowing a patient to share knowledge about their REA in a way that integrates it with other aspects of their background, family history, and healthcare experiences can lead to both clinical and interpersonal benefits (Nowaczyk, 2012). While we have argued against the routine practice of using REA information for risk assessment, establishing test eligibility, or guiding test selection, we do not wish to foreclose opportunities for patients to narrate personal stories related to their multiple identities in genetic counseling sessions. Our view is that it is unnecessary and potentially detrimental to structure these conversations in ways that imply patients could somehow describe their own identities incorrectly or in a manner that disqualifies them from receiving services. Rather, we believe facilitating the development of a patient's own identity narrative not only enriches the therapeutic relationship but also enhances the clinician's understanding of that patient's family, community, and systems of meaning and support.

Uplifting a patient's narrative experiences in genetic counseling encounters during which REA information is discussed can center meaningful insights and promote a more trusting therapeutic relationship. We suggest that genetic counselors approach gathering REA information in a way that respectfully explores how the patient's genetic information connects to their personal stories and cultural preferences. Questions to help guide this conversation might include: “Are there aspects of your cultural or familial traditions that you'd like me to know about to better support you?”, “What does it mean to you to have your story, culture, or background considered in your care?”, “Are there aspects of your community or cultural beliefs that are important for me to understand as we work together?” These questions might encourage patients to pause and examine if and how their knowledge about their race, ethnicity, or ancestry interacts with their medical information and health status. On the other hand, not all patients will feel comfortable responding to these proposed questions, or they may be uncertain about which parts of their identity are appropriate to share in a clinical environment. In all cases, it is important for the genetic counselor to assess the tone of the encounter and adjust their approach to align with the patient's comfort and knowledge levels. Some additional approaches to integrating narrative medicine components into genetic counseling encounters involve asking open‐ended questions, encouraging patients to discuss their worries or concerns, and actively learning who the patient is as a holistic person (Peterkin, 2012). Further, if genetic counselors are to encourage patients to carefully consider their racial, ethnic, and/or ancestral identi(ies), they must also themselves be reflective upon their own positionality and perspective on such topics. It is crucial for clinicians in all settings to identify their own biases and implicit assumptions they make about their patients if they are to authentically engage in narrative‐driven discussions.

## 6 USES OF REA INFORMATION IN VARIANT DISCOVERY AND HEALTH RESEARCH

By arguing that routinely collecting self‐reported REA information is not often useful in clinical practice, and further that it may be ethically problematic, we do not intend to undermine the broader value of these labels. Socially constructed categories have profound health consequences. REA information can provide valuable insight if collected and used purposefully and methodologically rigorously. Such constructs influence a person's experience with discrimination, access to resources, and social networks. Because these dimensions of identity are linked to health outcomes, self‐reported REA data can offer critical insight for researchers investigating the social determinants of health and health disparities (Borrell et al., 2021). Self‐reported identities reflect how individuals navigate their sociocultural surroundings, including their experiences with discrimination, access to resources, and social networks. These factors play a central role in health outcomes. Accordingly, it is entirely appropriate to continue using self‐reported REA labels in research on the social determinants of health and health disparities, provided that care is taken to report these data in ways that do not imply that population groups widely differ due to biological traits.

We also recognize that limiting the collection of REA information in clinical settings may also restrict opportunities for crucial health disparities research and health policy improvements, much of which relies on data gathered in clinical settings. This use of REA information does not justify uncritical data collection. The ethical imperative must be to protect principles of autonomy, justice, and respect for privacy. Health, identity, and genetic data are not themselves neutral; they are embedded in broader relationships of power and responsibility. Rather than defaulting to routine REA collection for the sake of gathering additional clinical variables, we advocate for practices that acknowledge and uphold the relational and identity‐based nature of these data. Further research should explore how clinicians collect and use REA information, the extent to which existing guidelines shape practice, whether relevant ethical considerations are incorporated into clinical education, and how patients themselves perceive and understand discussions about REA in genetic counseling encounters.

## 7 CONCLUSION

It is crucial to balance the clinical benefits of gathering REA information from patients with the ethical, social, and interpersonal implications of doing so. The complex interactions between genetics, ancestry, and identity help to tell part of the story of who patients are, and providers should not expect them to leave this behind by foregoing conversations about REA altogether. It is imperative that healthcare providers, specifically those engaging with genetics and genomics, are equipped to facilitate patient‐led conversations about REA during clinical encounters. Embracing narrative medicine may allow genetic counselors to facilitate meaningful conversations about REA that respect the patient's preferences, experiences, and background so that professionals are focusing not on what services patients qualify for, but who they are and how they choose to identify.

## AUTHOR CONTRIBUTIONS

**Emily Peugh:** Conceptualization; writing – original draft; writing – review and editing. **Sara Chandros Hull:** Conceptualization; writing – review and editing. **Leila Jamal:** Conceptualization; writing – original draft; writing – review and editing.

## DISCLAIMER

This research was supported [in part] by the Intramural Research Program of the National Institutes of Health (NIH). The contributions of the NIH author(s) were made as part of their official duties as NIH federal employees, are in compliance with agency policy requirements, and are considered Works of the United States Government. However, the findings and conclusions presented in this article are those of the author(s) and do not necessarily reflect the views of the NIH or the U.S. Department of Health and Human Services.
